# Hormonome Dynamics During Microgametogenesis in Different *Nicotiana* Species

**DOI:** 10.3389/fpls.2021.735451

**Published:** 2021-10-15

**Authors:** Lenka Záveská Drábková, Eva Pokorná, Petre I. Dobrev, Jana Kůrková, Lenka Steinbachová, David Honys, Václav Motyka

**Affiliations:** ^1^Laboratory of Pollen Biology, Institute of Experimental Botany of the Czech Academy of Sciences, Prague, Czechia; ^2^Laboratory of Hormonal Regulations in Plants, Institute of Experimental Botany of the Czech Academy of Sciences, Prague, Czechia

**Keywords:** hormonome, male gametophyte, *Nicotiana* spp., ontogeny, pollen development, phytohormones

## Abstract

Plant microgametogenesis involves stages leading to the progressive development of unicellular microspores into mature pollen. Despite the active and continuing interest in the study of male reproductive development, little is still known about the hormonomics at each ontogenetic stage. In this work, we characterized the profiles and dynamics of phytohormones during the process of microgametogenesis in four *Nicotiana* species (*Nicotiana tabacum*, *Nicotiana alata*, *Nicotiana langsdorffii*, and *Nicotiana mutabilis*). Taking advantage of advanced HPLC-ESI-MS/MS, twenty to thirty endogenous hormone derivatives were identified throughout pollen ontogenesis, including cytokinins, auxins, ABA and its derivatives, jasmonates, and phenolic compounds. The spectra of endogenous phytohormones changed dynamically during tobacco pollen ontogeny, indicating their important role in pollen growth and development. The different dynamics in the accumulation of endogenous phytohormones during pollen ontogenesis between *N. tabacum* (section *Nicotiana*) and the other three species (section *Alatae*) reflects their different phylogenetic positions and origin within the genus *Nicotiana*. We demonstrated the involvement of certain phytohormone forms, such as *cis*-zeatin- and methylthiol-type CKs, some derivatives of abscisic acid, phenylacetic and benzoic acids, in pollen development for the first time here. Our results suggest that unequal levels of endogenous hormones and the presence of specific derivatives may be characteristic for pollen development in different phylogenetic plant groups. These results represent the currently most comprehensive study of plant hormones during the process of pollen development.

## Introduction

The development of male gametophyte in vascular plants is a complex process that requires coordinated action and participation of numerous cells and both sporophyte and gametophyte tissues. The life cycle of the male gametophyte can be divided into a developmental phase leading to the formation of mature pollen grains and a functional (progamic) phase beginning with the impingement of pollen on the stigma surface and ending with double fertilization (Hafidh and Honys, 2021).

The pollen grain of angiosperms is formed within the anthers of the flower via two successive developmental programs: microsporogenesis and microgametogenesis. During microsporogenesis, one (lineage model, Scott et al., 2004) to a few (cluster model, Kelliher and Walbot, 2011) meristematic L2-derived sporophytic cells differentiate into archesporial cells, which will undergo a series of periclinal and anticlinal divisions giving rise to the sub-epidermal anther wall layers (endothecium, middle layer, and tapetum) and the pollen mother cells. The diploid pollen mother cells will then undergo meiosis and form tetrads of haploid microspores encased by a callose layer. The digestion of callose and the release of microspores mark the beginning of microgametogenesis. Microgametogenesis comprises events that lead to the progressive development of the unicellular microspores into mature pollen. The released microspores increase their size, vacuolize, and their nuclei migrate to the periphery (McCormick, 1993; Borg and Twell, 2010). Thereafter, the microspores undergo highly asymmetric pollen mitosis I (PMI), leading to a large vegetative cell and a small generative cell. The generative cell migrates into the cytoplasm of the vegetative cell (Berger and Twell, 2011). After PMI, the generative cell moves inward, enabling gamete transport within the pollen tube (Honys et al., 2006). In *Nicotiana*, the generative cell undergoes one more cell division, pollen mitosis II (PMII), which occurs before pollen maturation in bi-cellular pollen. Two sperm cells are formed from a generative cell by PMII. The fusion of one sperm cell with the egg cell results in the formation of embryo, and the other sperm cell’s fusion with the central cell initiates the endosperm, a nutritive tissue for the embryo. The endosperm and double fertilization are unique and are often used as defining features of angiosperms.

An essential role in male gametophyte development is played by phytohormones -naturally occurring low-abundance organic substances affecting numerous physiological processes in plants such as germination, seed development, vegetative growth, flowering, senescence, dormancy, mobilization of nutrients as well as the biotic and abiotic stress responses (Novák et al., 2017; Kieber and Schaller, 2018; Wybouw and de Rybel, 2019). Based on their physiological functions and chemical structures, phytohormones are classified into several groups – auxins, cytokinins (CKs), gibberellins (GAs), abscisic acid (ABA), ethylene, polyamines, brassinosteroids (BRs), jasmonates, salicylic acid (SA) plus other hormones with a phenolic nature, signaling peptides and strigolactones (Novák et al., 2017; Šimura et al., 2018). Hormonal studies of the plant microgametogenesis have been mostly limited to the effects of exogenously applied phytohormones on various aspects of pollen development (Kovaleva et al., 2015; Zuñiga-Mayo et al., 2018), their immunolocalization (Chen and Zhao, 2008), and molecular regulations (Huang et al., 2003; Chhun et al., 2007; Cecchetti et al., 2008, 2015, 2017; Kinoshita-Tsujimura and Kakimoto, 2011; Ding et al., 2012; Yao et al., 2018).

However, systematic research on the profiles of various phytohormone classes and their dynamics throughout particular pollen ontogenetic stages has not been performed yet, and the existing data are scarce and scattered. Chibi et al. (1995) reported variations in the ABA level during pollen grains maturation in *Nicotiana tabacum*, whereas changes in the endogenous auxin concentrations were sketched by Dupl’áková et al. (2016) for five pollen developmental stages in *Arabidopsis thaliana.* Indirect evidence for a high IAA accumulation in the *Arabidopsis* pollen grains was provided by Salinas-Grenet et al. (2018) in transgenic plants expressing DR5::GUS promoter. A strong auxin signaling activity was detected throughout the pollen development, mainly in the uninucleate microspore, bicellular and tricellular cells. The endogenous profile of several phytohormones in the anther, leaf blade and pistil of rice was presented by Hirano et al. (2008). A marked predominance of free IAA, IAA-aspartate (IAA-Asp) and GA4, in contrast to very low levels of CK derivatives, was shown in anthers, containing mainly mature tricellular pollen grains.

Hirano et al. (2008) have given an extensive picture of the involvement of endogenous phytohormones in the microspore/pollen development. It is, however, not possible to generalize Hirano’s results on rice monocotyledonous plant model for dicotyledonous plants. That’s why we focused here on determining variations in the plant hormonome during microgametogenesis in tobacco as a dicotyledonous plant representative. Taking advantage of advanced high-performance liquid chromatography tandem mass spectrometry, we provide a detailed analysis of endogenous phytohormones during pollen ontogeny in four tobacco species differing in their phylogenetic position within the genus *Nicotiana*, the model plant *N. tabacum* (section *Nicotiana*) and three other species (*Nicotiana alata, Nicotiana langsdorffii*, and *Nicotiana mutabilis*; all section *Alatae*). The obtained results represent the currently most comprehensive overview of the plant hormonome during the process of pollen development in plants.

## Materials and Methods

### Plant Material and Growth Conditions

**FIGURE 1:**
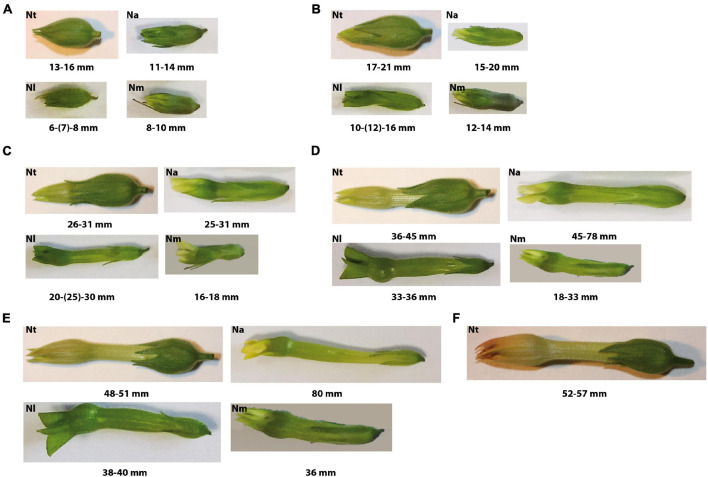
Shape and size of floral buds containing main pollen developmental stages of genus *Nicotiana*: **(A)** Stage 1 – unicellular microspores; **(B)** Stage 2 – early bicellular young vacuolized pollen grains with beginning starch deposition in *N. alata* (Na), *N. langsdorffii* (Nl) and *N. mutabilis* (Nm) and early bicellular young vacuolized pollen in *N. tabacum* (Nt); **(C)** Stage 3 – middle bicellular pollen in Na, Nl, and Nm and early bicellular pollen with beginning starch deposition in Nt; **(D)** Stage 4 – late bicellular pollen with spindle-shaped generative nucleus in Na, Nl, Nm, and middle bicellular pollen filled with starch in Nt; **(E)** Stage 5 – mature pollen grain in Na, Nl, Nm, and late bicellular pollen with spindle-shaped generative nucleus in Nt; **(F)** Stage 6 – mature pollen grain in Nt.

All developmental pollen stages of *Nicotiana tabacum* L. were separately collected during July 2019 and 2020 on the basis of the length of the flower buds including calyx and corolla (Tupý et al., 1983). For *Nicotiana tabacum*, six main stages were obtained: stage 1 (unicellular microspores; 1-UNC) – 13–16 mm; stage 2 (early bicellular pollen; 2-early BC) – 17–21 mm, stage 3 (3-BC) – 26–31 mm, stage 4 (4-BC) – 36–45 mm, stage 5 (late bicellular pollen; 5-BC) – 48–51 mm, and stage 6 (mature pollen grain; MPG) – 52–57 mm. We also collected flower buds with different male gametophyte developmental stages from *N. alata* Link & Otto, *N. langsdorffii* Weinm., and *N. mutabilis* Stehmann & Semir during a whole season and checked them microscopically to select clearly definable stages. Selected developmental stages of *N. alata*, *N. langsdorffii*, and *N. mutabilis* were compared with *N. tabacum.* Only five developmental stages were detected and retained (Figure 1). Flower bud lengths and morphology were measured and determined under a Zeiss stereomicroscope (Carl Zeiss, Jena, Germany).

Freshly isolated anthers from 20 flower buds of particular developmental stage (Tupý et al., 1983) were collected and immediately processed by gentle crushing in a chilled mortar within a 5% sucrose solution. The mixture was vortexed immediately for 20 s to release the pollen grains and filtered through a nylon mesh (approximately 100 μm of pore size) to remove anther debris. The suspended pollen grains were then sedimented by centrifugation (2,000 *g*, 5 min, 4°C), and after decanting excess supernatant were stored at −80°C as described by Matoušek et al. (2020).

Mature pollen grains (MPG) from isolated anthers of flowers 1 day before anthesis (Tupý et al., 1983; Matoušek et al., 2020) were collected daily and were desiccated on filter paper in Petri dish to dehisce overnight at room temperature. Then, MPG were sieved through a nylon mesh to discard anther debris and stored at −80°C.

### Pollen Microscopy

**FIGURE 2:**
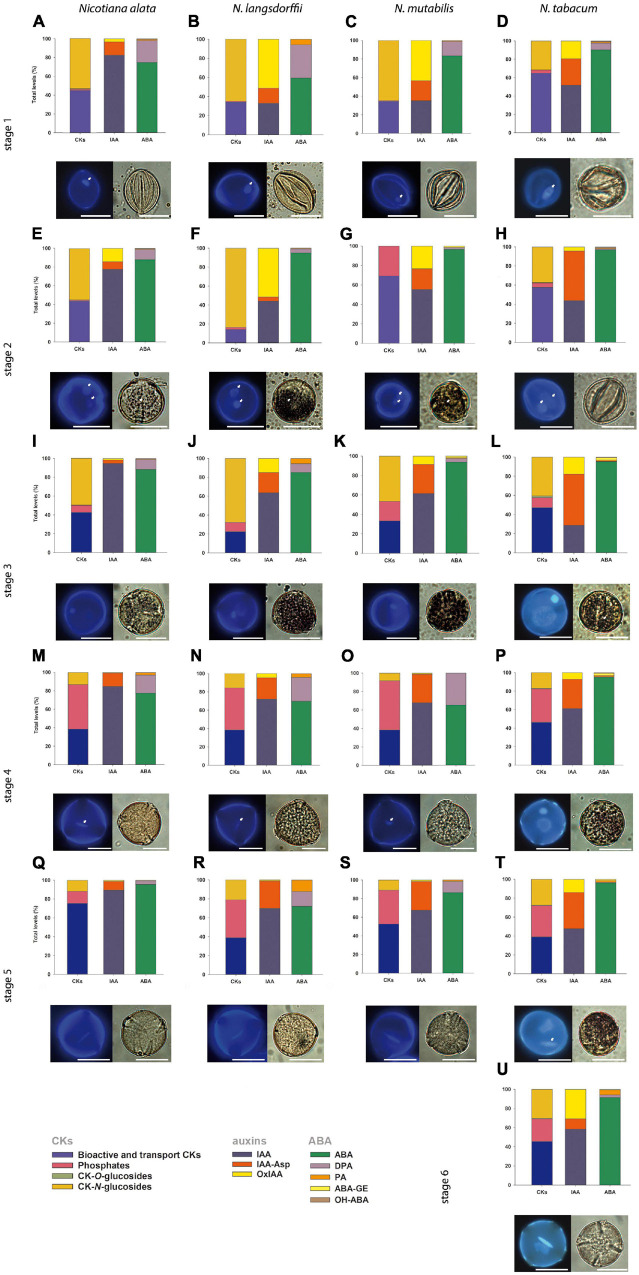
General schematic overview demonstrating the shape of nuclei, starch content and the profiles of phytohormones cytokinins, (CKs), auxins (indole-3-acetic acid, IAA) and abscisic acid (ABA) (together with their derivatives; for specification see lower-left corner of the panel) during pollen development of *Nicotiana alata* **(A,E,I,M,Q)**, *N. langsdorffii* **(B,F,J,N,R)**, *N. mutabilis* **(C,G,K,O,S)**, and *N. tabacum* **(D,H,L,P,T,U)**. The scale is 20 μm. **(A–D)** Stage 1, **(E–H)** stage 2, **(I–L)** stage 3, **(M–P)** stage 4, **(Q–T)** stage 5, **(U)** stage 6. Characterization of individual pollen developmental stages is given in Figure 1, proportions of CKs, auxins, and ABA are expressed as a percentage of the total pool. Small white arrows show beginning of starch deposition at stage 2 **(E,F,G)** and at stage 3 **(L)**; one nucleolus in microspores **(A–D)** and generative and vegetative nuclei starting at stage 2 **(E–H)**; and spindle-shaped generative nucleus at stage 4 **(M–O)** and at stage 5 **(T)**.

Small aliquots of *Nicotiana* spp. pollen of different stages (0.5–1 μL) were resuspended in DAPI staining solution (4′-6-Diamidino-2-phenylindole, Merck KGaA, Darmstadt, Germany) for cell nuclei visualization [16 μL of DAPI stock solution in 10 mL buffer, modified according to Dupl’áková et al. (2016)] and Lugol’s staining solution for starch grains visualization (3.5% solution: 5 g KI, 2.5 g I, 200 ml 80% ethanol). Bright field and fluorescence (UV light) microscopy were used for checking the particular stage of tobacco pollen and starch content on an inverted fluorescent microscopes Nikon Eclipse TE2000E and Zeiss Axiovert 200M. NIS-Elements (Nikon Imaging Software) and ZEN blue 3.2 (Carl Zeiss, Jena, Germany) software were used to capture images. For each pollen stage, representative images illustrating the shape of nucleus and the starch content are shown (Figure 2).

### Phytohormone Extraction and Quantification

Endogenous phytohormones were extracted according to Dobrev and Kamínek (2002). Homogenized samples [approximately 100 mg fresh weight (FW)] were incubated in 0.5 mL Bielski extraction buffer (methanol/formic acid/water 15/1/4, v/v/v) with addition of internal standards (10 pmols each) for 1 h at −20°C. After centrifugation (20,000 *g*, 25 min, 4°C), the pellets were re-extracted with an additional 0.5 mL of Bielski buffer for 30 min at 6°C. Samples were concentrated in a vacuum concentrator (Alpha RVC, Christ, Germany), diluted with 0.5 mL of 1 M formic acid and applied to a mixed-mode reversed phase-cation exchange SPE column (Oasis-MCX, Waters, Milford, MA, United States). Two fractions were obtained: (1) fraction A (eluted with 0.5 mL methanol) containing hormones of acidic and neutral character (IAA, ABA, jasmonates and phenolic compounds); and (2) fraction B (eluted with 0.5 mL 0.35 M NH_4_OH in 60% methanol) containing hormones of basic character (CKs). The SPE eluates were evaporated to dryness in a vacuum concentrator. The fraction A was then dissolved in 30 μL 15% of acetonitrile and the fraction B in 30 μL 5% of methanol. An aliquot (10 μL) of each extract was injected on an Ultimate 3000 high-performance liquid chromatograph (Dionex, Bannockburn, IL, United States) coupled to a hybrid triple quadrupole/linear ion trap mass spectrometer (3200 Q TRAP, Applied Biosystems, Foster City, CA, United States) using a multilevel calibration graph with [^2^H]-labeled internal standards as described previously Dobrev and Vaňková (2012) and Djilianov et al. (2013). Methylthiolated *cis*- and *trans-*zeatin-type CKs (ribosides of *c*Z and *t*Z) were not distinguishable by HPLC-MS due to identical mass spectra and are therefore considered one substance marked in the graph legend as 2MeSZR. Phytohormone levels were recalculated to dry weight because the water content substantially differed during pollen maturation (from 13.25% in mature pollen grains up to 86.2% during pollen development).

## Results

### Developmental Changes and Starch Accumulation During Pollen Ontogeny of Different *Nicotiana* Species

In *Nicotiana tabacum* (section *Nicotiana*), six stages of pollen development have previously been identified and distinguished on the basis of bud length, flower size and morphology, organelle development, and starch content (Tupý et al., 1983). In contrast, we determined only five developmental stages in *N. alata, N. langsdorffii*, and *N. mutabilis* (section *Alatae*). The variation in flower bud morphology and sizes of all sampled stages (Figure 1) were ascertained to the corresponding pollen developmental stage (Figure 2). Stage 1 of *Nicotiana tabacum* is characterized by unicellular, mostly polarized microspores before pollen mitosis I (PMI). Early bicellular young vacuolized pollen (stages 2) and early bicellular pollen with beginning starch deposition (stage 3) to middle bicellular pollen (stage 4) contain two nuclei, one generative and one vegetative. The generative nucleus of late bicellular pollen in premature stage 5 begins to elongate in a spindle shape. Stage 6 is represented by a mature pollen grain before anthesis (Figure 2). Five main developmental stages were found in the other three species, which probably reflects a faster developmental process during the first mitosis of the microspore (Figure 2). Stage 1 is the same as in *N. tabacum*, a unicellular microspore, but the two-celled pollen stage contains two stages of bicellular pollen described as early bicellular pollen with beginning starch deposition and middle bicellular pollen (stages 2 and 3, respectively), followed by elongation and spindle-like formation of the generative nucleus at stage 4 – late bicellular pollen, and stage 5 is the mature pollen just before anthesis.

Due to the evolutionary tendency in higher plants to store material reserves and nutrients during pollen maturation, we visualized starch content in individual *Nicotiana* pollen stages. Together with increasing flower size, the progression of pollen development from microspores to the late bicellular pollen stage and starch accumulation (Figure 2) between stages 2 and 5 were found to be species-dependent. In *N. tabacum*, starch accumulation does not occur at stage 2, while it becomes evident at stage 3 and peaks at stage 4. A high starch content is still detectable at stage 5 but not at stage 6. In *N. alata, N. langsdorffii*, and *N. mutabilis*, starch accumulation occurs more rapidly (starch grains are already visible at stage 2) and reaches maxima at stage 3 (Figure 2).

### Hormonal Profiling During the Pollen Development in Different *Nicotiana* Species

**FIGURE 3:**
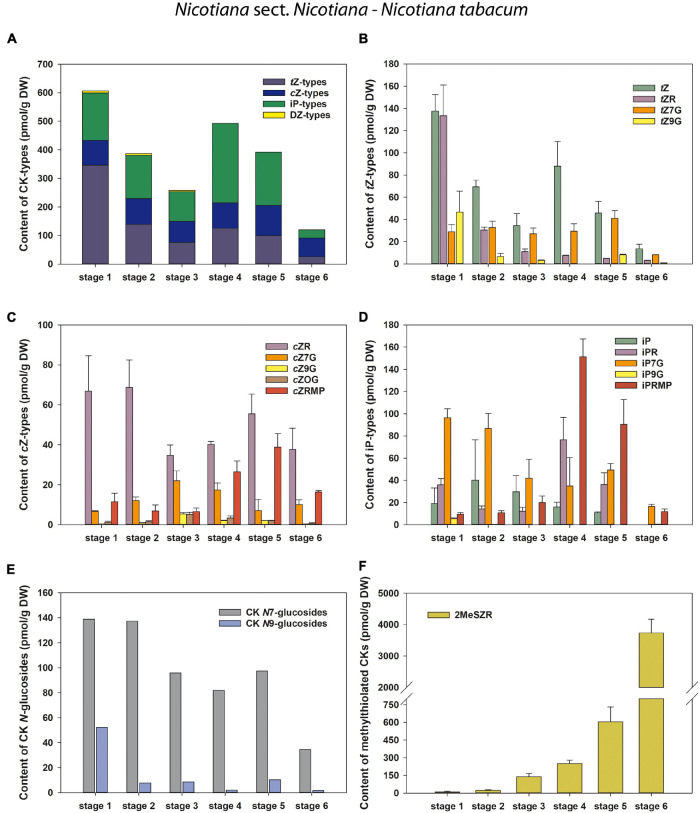
Changes in the content of endogenous cytokinins (CKs) during six developmental stages of *Nicotiana tabacum* pollen. **(A)** Total CKs; **(B)** *trans*-zeatin (*t*Z) types; **(C)** *cis*-zeatin (*c*Z) types; **(D)** *N*^6^-(Δ^2^-isopentenyl)adenine (iP) types; **(E)** CK *N*7- and *N*9-glucosides; **(F)** Methylthiol-types (represented by 2MeSZR).

**FIGURE 4:**
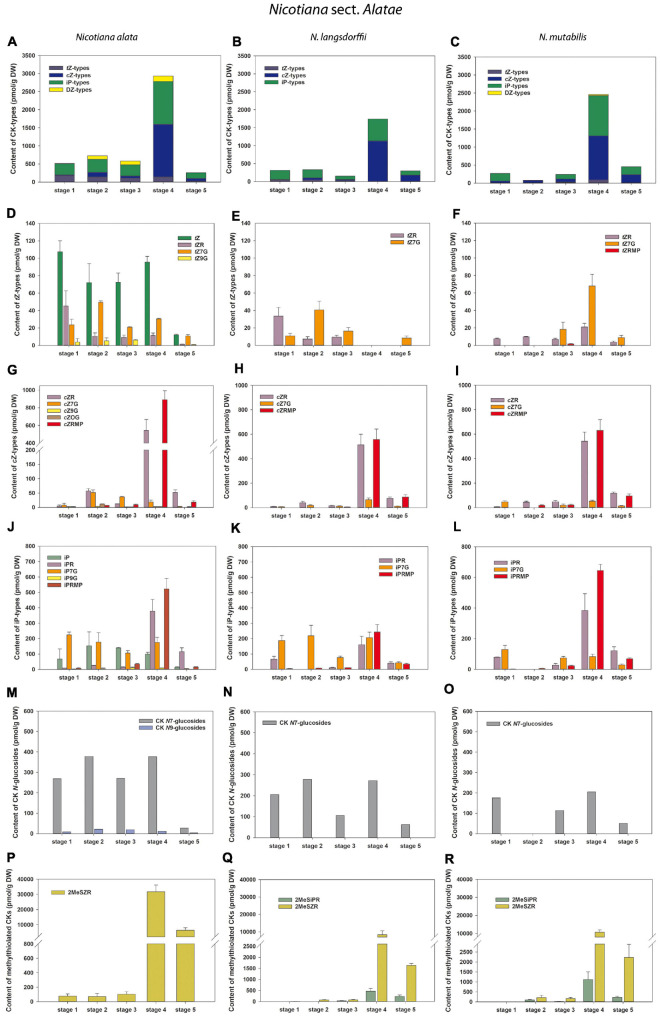
Changes in the content of endogenous cytokinins (CKs) during five developmental stages of *Nicotiana alata* **(A,D,G,J,M,P)**, *N. langsdorffii* **(B,E,H,K,N,Q)**, and *N. mutabilis* **(C,F,I,L,O,R)**. **(A–C)** Total CKs; **(D–F)** *trans*-zeatin (*t*Z) types; **(G–I)** *cis*-zeatin (*c*Z) types; **(J–L)** *N*^6^-(Δ^2^-isopentenyl)adenine (iP) types; **(M–O)** CK *N*7- and *N*9-glucosides; **(P–R)** Methylthiol-types (represented by 2MeSZR and 2MeSiPR).

**FIGURE 5:**
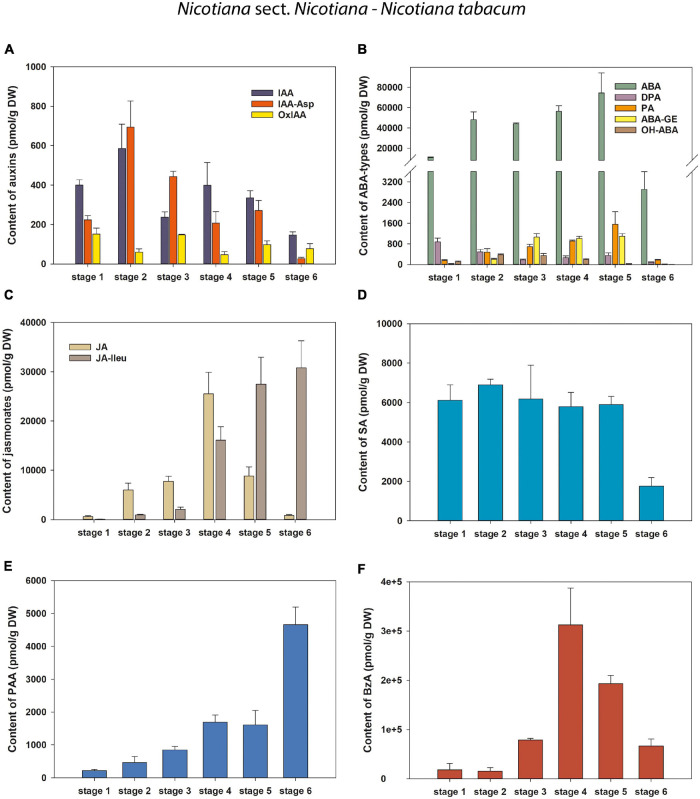
Changes in the content of endogenous auxins, ABA and its derivatives, jasmonates and phenolic compounds during six developmental stages of *Nicotiana tabacum* pollen. **(A)** Auxins (IAA, indole-3-acetic acid; IAA-Asp, IAA-aspartate; OxIAA, 2-oxindole-3-acetic acid); **(B)** Abscisic acid (ABA) types [DPA, dihydrophaseic acid; PA, phaseic acid; (ABA-GE), ABA glucosyl ester; OH-ABA, 9-hydroxy-ABA]; **(C)** Jasmonates (JA, jasmonic acid; JA-Ileu, JA-isoleucine); **(D–F)** Phenolic compounds (**D** SA, salicylic acid; **E** PAA, phenylacetic acid; **F** BzA, benzoic acid).

**FIGURE 6:**
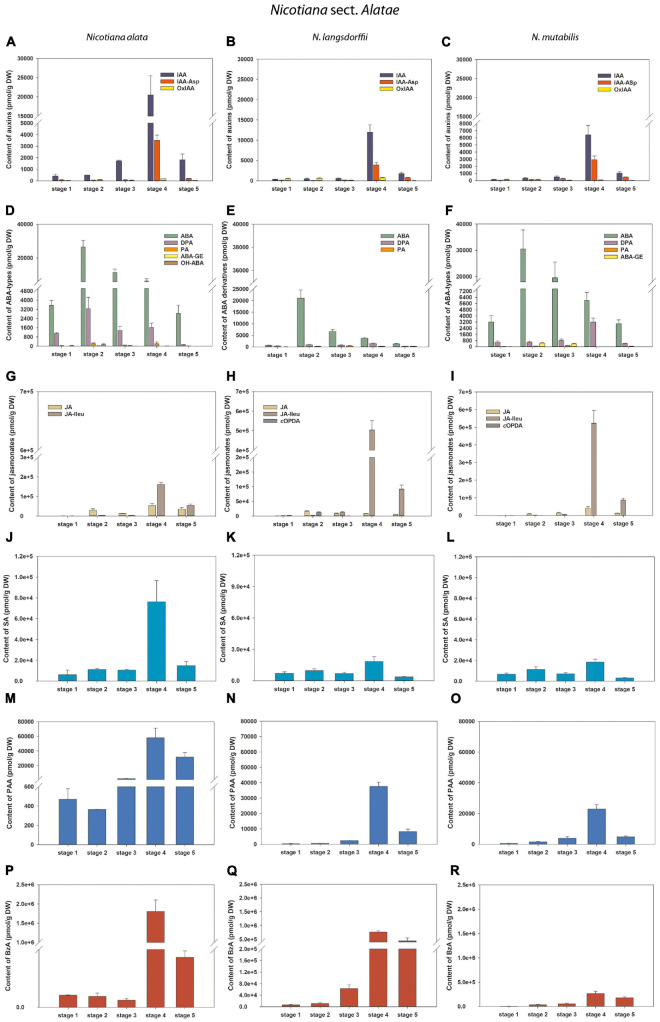
Changes in the content of endogenous auxins, ABA and its derivatives, jasmonates and salicylic acid during five developmental stages of *Nicotiana alata* **(A,D,G,J,M,P)**, *N. langsdorffii* **(B,E,H,K,N,Q)**, and *N. mutabilis* **(C,F,I,L,O,R)** pollen. **(A–C)** Auxins (IAA, indole-3-acetic acid; IAA-Asp, IAA-aspartate; OxIAA, 2-oxindole-3-acetic acid); **(D–F)** Abscisic acid (ABA) types [DPA, dihydrophaseic acid; PA, phaseic acid; (ABA-GE), ABA glucosyl ester; OH-ABA, 9-hydroxy-ABA]; **(G–I)** Jasmonates [JA, jasmonic acid; JA-Ileu, JA-isoleucine; *c*OPDA, *cis*-(+)-12-oxo-phytodienoic acid]; **(J–L)** Salicylic acid (SA); **(M–O)** Phenylacetic acid (PAA); **(P–R)** Benzoic acid (BzA).

**FIGURE 7:**
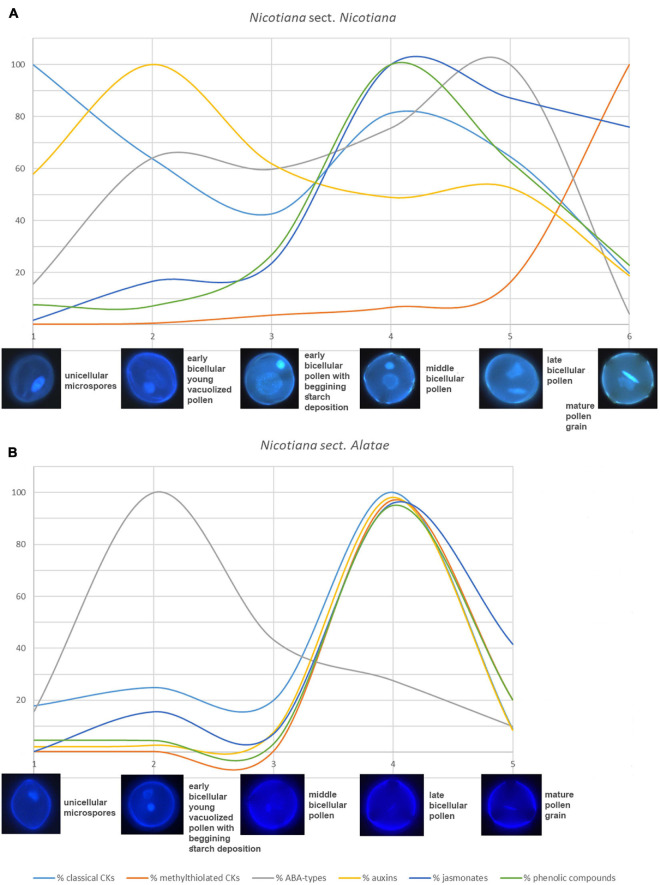
General summary of changes and schematic representation of different trends in all analyzed phytohormone levels between **(A)** *Nicotiana* sect. *Nicotiana* (*Nicotiana tabacum*) and **(B)** *Nicotiana* sect. *Alatae* (*N. alata, N. langsdorffii*, and *N. mutabilis* that showing same trends). Different colors represent percentage of particular phytohormones [light blue – classical cytokinins, red – methylthiolated cytokinins, gray – abscisic acid (ABA) types, yellow – auxins, dark blue – jasmonates, green – phenolic compounds].

Hormone analysis was performed after dual-mode solid-phase extraction and using an advanced high-performance liquid chromatography tandem mass spectrometry. This procedure allowed the simultaneous and highly reliable identification and quantification of nearly thirty phytohormones, including CKs, auxins, ABA, JA (all together with their various metabolic forms), and the phenolic compounds SA, benzoic acid (BzA), and phenylacetic acid (PAA). The concentrations of these different phytohormones varied considerably within six ontogenetic stages of *Nicotiana tabacum* and five ontogenetic stages of *N. alata, N. langsdorffii*, and *N. mutabilis*, as shown in Figures 3–7 and Supplementary Tables 1–4.

A general schematic overview of the profiles of growth hormones (CKs and auxins) and the most abundant stress hormone ABA (together with its derivatives) during pollen development in different *Nicotiana* species is shown in Figure 2. Based on the criteria of biological function/conjugation status, the classical CKs (i.e., excluding methylthiolated derivatives) are classified here into four groups, including (1) bioactive and transport forms (free bases and ribosides, respectively), (2) immediate biosynthetic precursors (phosphates), (3) storage forms (*O*-glucosides), and (4) deactivation forms (*N-*glucosides). Although it is rather difficult to generalize variations in the proportion of particular CK forms in the total CK pool during pollen ontogeny, some clues are worth considering. These include relatively high levels of bioactive and transport CK forms (on average over 40% of total CKs) and the absence or lack of CK *O*-glucosides (less than 0.1% of the total CKs) throughout pollen development in all four species, the abundance of CK phosphates at later developmental stages (on average 48% of total CKs) and their low levels at early stages (6% of total CKs) in all species (except at stage 2 in *N. mutabilis*) and a decreasing trend in the concentrations of CK *N*-glucosides during pollen ontogeny in *N. alata* and *N. langsdorffii* (Figure 2 and Supplementary Tables 1–4) are among the most important.

During pollen development only a relatively narrow spectrum of endogenous indole auxins was detected, including bioactive free indole-3-acetic acid (IAA), its major primary catabolite 2-oxindole-3-acetic acid (OxIAA) and its amino acid conjugate IAA-aspartate (IAA-Asp; Figure 2 and Supplementary Tables 1–4). Among indole auxins, IAA dominated throughout pollen ontogeny in *N. alata* (averaging over 85% of the total) and at later developmental stages in all other three species. Concentrations of IAA-Asp exceeded those of OxIAA during pollen development of *N. tabacum* (except at stage 6) and at later developmental stages (stages 3–5) of *N. alata*, *N. langsdorffii*, and *N. mutabilis.*

The total pool of ABA metabolites comprised from three (*N. langsdorffii*) to five (*N. alata*, *N. tabacum*) substances, including bioactive ABA itself (Figure 2 and Supplementary Tables 1–4). In all four species, ABA was the predominant form throughout pollen development, accounting for between 86% (*N. alata*) and 96% (*N. tabacum*) of the total pool. Derivatives of ABA accounted for the remaining proportion (from 4 to 14%). Among them, the deactivation product dihydrophaseic acid (DPA) predominated throughout pollen ontogeny in *N. alata*, *N. langsdorffii*, and *N. mutabilis*. In *N. tabacum*, DPA represented the major ABA metabolite at stage 1, while the other catabolite phaseic acid (PA) and the storage form ABA glucosyl ester (ABA-GE) prevailed in later stages (Figure 2 and Supplementary Tables 1–4).

To summarize, our data suggest that CKs, auxins, and ABA derivatives persist predominantly in bioactive forms throughout pollen ontogeny in all four tobacco species. The levels of bioactive forms, similar to the levels of other metabolites, change dynamically during each developmental stage. This suggests that phytohormones are integrally involved in and rigorously influence the growth and developmental processes of tobacco pollen.

### Variations in Phytohormones Contents During Pollen Ontogeny of Different *Nicotiana* Species

#### Cytokinins

A total of eight to fifteen forms of four classical isoprenoid CKs –*N*^6^-(Δ^2^-isopentenyl)adenine (iP), *trans-*zeatin (*t*Z), *cis-*zeatin (*c*Z), and dihydrozeatin (DZ) – were detected during the course of pollen development in individual tobacco species (Figures 3, 4 and Supplementary Tables 1–4). Concentrations of each CK derivative varied in tobacco pollen from picomoles to hundreds of picomoles per g DW. Interestingly, no CK *O*-glucosides (storage forms of CKs) or only their trace amounts near the detection limit (less than 0.1% of total CKs) were found. In addition to classical CKs, CK methylthioderivatives of zeatin-type (ribosides of *c*Z and *t*Z; designated as 2MeSZR) and iP-type (riboside of iP; 2MeSiPR) occurred in pollen at high concentrations that substantially exceeded the levels of other CKs, especially at later developmental stages (Figures 3, 4 and Supplementary Tables 1–4).

In *N. tabacum*, the total pool of classical CKs in developing pollen grains was composed of *t*Z (four forms), *c*Z, iP (five forms each), and DZ (one form) derivatives (Figure 3 and Supplementary Table 1). Total concentrations of CKs varied during pollen ontogeny from 119.86 (mature pollen grains; stage 6) to 606.21 pmol/g DW (microspores; stage 1). In addition to the CK maximum in microspores, another peak of CKs was detectable in bicellular pollen grains (stage 4) (Figure 3A). The first and most important peak observed at stage 1 in microspores was mainly caused by the maxima of *t*Z-type CKs. Among them, *t*Z and its riboside (*t*ZR) dominated, both appearing at concentrations above 130 pmol/g DW (Figure 3B). The iP-types represented the most abundant CKs at later developmental stages, with the exception of stage 6, where the *c*Z-types predominated (Figures 3C,D). The second (smaller) maximum of total CKs in *N. tabacum* pollen, detected at stage 4 (493.38 pmol/g DW) can be explained by an increase in *N*^6^-(Δ^2^-isopentenyl)adenosine-5′-monophosphate (iPRMP) level (151.31 pmol/g DW), followed by *t*Z and *N*^6^-(Δ^2^-isopentenyl)adenosine (iPR). The *c*Z-type CKs were present at moderate levels during the ontogeny of *N. tabacum* pollen (from 65.23 to 105.5 pmol/g DW). In contrast, the sole representative of the DZ-types, DZ-*N*7-glucoside (DZ7G), occurred only in very low amounts in the first three developmental stages (below 7 pmol/g DW) and was not detected in later ones. The content of *N*7-glucosides significantly exceeded that of *N*9-glucosides throughout pollen development (3- to 40-fold) (Figure 3E). Interestingly, a progressively increasing accumulation of 2MeSZR was detected during ontogeny of *N. tabacum* pollen (Figure 3F).

Total concentrations of CKs in the other three tobacco species varied considerably during pollen development, ranging from 77.84 (*N. mutabilis*, stage 2) to 2933.43 pmol/g DW (*N. alata*, stage 4) (Figure 4 and Supplementary Tables 2–4). In general, *c*Z- and iP-type CKs predominated, followed by *t*Z-types. Similar to *N. tabacum*, the DZ-types represented the least abundant or absent CK forms (Figures 4A–C). The total CK pool in the pollen of all three tobacco species reached the characteristic maxima at stage 4, mainly caused by the peaks of the bioactive and phosphate *c*Z and iP forms (Figures 4D–L). The spectra of classical CKs in pollen differed among tobacco species and included eight (*N. langsdorffii*), ten (*N. mutabilis*), and fifteen (*N. alata*) derivatives. In analogy to *N. tabacum*, *N*7-glucoside concentrations in *N. alata* significantly exceeded *N*9-glucosides (7- to 35-fold) during pollen development, whereas CK *N*9-glucosides were completely absent in *N. langsdorffii* and *N. mutabilis* pollen (Figures 4M–O and Supplementary Tables 2–4). Accumulation of methylthiolated CKs (2MeSZR in *N. alata* and both 2MeSZR and 2MeSiPR in *N. langsdorffii* and *N. mutabilis*) had a similar pattern to classical CKs, with maxima at stage 4 (Figures 4P–R).

Overall, CK screening during pollen development revealed some trends for classical and methylthiolated CKs that differ between *N. tabacum* and the other three (*N*. *alata*, *N. langsdorffii*, and *N. mutabilis*) species. Among them, two concentration maxima of the total pool of classical CKs (stages 1 and 4) in *N. tabacum* pollen compared to one peak (stage 4) in the other three species and/or the progressive increase in the content of CK methylthioderivatives during the ontogeny of *N. tabacum* pollen compared to. their maximum accumulation at stage 4 in the other three species are worth mentioning and deserve further attention.

#### Auxins

Only three endogenous auxins (IAA, OxIAA, and IAA-Asp) were found during pollen ontogeny in all four tobacco species (Figures 5, 6 and Supplementary Tables 1–4). In *N. tabacum* pollen, total auxin concentrations ranged from 250.52 (mature pollen grains; stage 6) to 1338 pmol/g DW (early bicellular pollen; stage 2). The auxin peak at stage 2 was mainly due to the IAA and IAA-Asp concentration maxima (584.47 and 693.94 pmol/g DW, respectively; Figure 5A). In the other tobacco species, the dynamics of total auxin accumulation were similar to those of CKs, with maxima at stage 4 (from 9459.23 in *N. mutabilis* to 24171.9 pmol/g DW in *N. alata*) caused in particular by IAA itself (Figures 6A–C). Interestingly, the highest IAA concentrations in pollen of all three species at stage 4 essentially overlapped the IAA maximum observed in *N. tabacum* at stage 2 (11-fold in *N. mutabilis* to 35-fold in *N. alata*). The concentrations of PAA, a phenolic non-indole compound with weak auxin activity, considerably exceeded those of indole auxins and showed analogous dynamics with the maximum at stage 4 in *N. alata*, *N. langsdorffii*, and *N. mutabilis* (Figures 5E, 6M–O).

To conclude, different dynamics of auxin accumulation were observed in the pollen of selected tobacco species, peaking at the earlier stage of development (stage 2) in *N. tabacum* and at the later stage (stage 4) in the other three species.

#### Stress Hormones (Abscisic Acid, Jasmonates)

Abscisic acid was present at relatively high concentrations throughout pollen development of all four tobacco species, ranging from 663.72 (*N. langsdorffii* at stage 1) to 74563.27 pmol/g DW (*N. tabacum* at stage 5) (Figures 5, 6 and Supplementary Tables 1–4). In *N. tabacum* pollen, an increasing trend of ABA content until stage 5 and its subsequent dramatic decrease at stage 6 (over 25-fold) were observed (Figure 5B). In the other three species, the ABA contents reached the concentration maxima at stage 2 (between 21028.12 and 30476.93 pmol/g DW in *N. langsdorffii* and *N. mutabilis*, respectively), and then decreased (9- to 16-fold) until stage 5 (Figures 6D–F).

In addition, several ABA derivatives were detected during tobacco pollen ontogeny. These included the deactivation products DPA and PA (in all four species), the storage form ABA-GE (except in *N. langsdorffii*) and another physiologically inactive ABA catabolite, 9-hydroxy-ABA (9OH-ABA) (only in *N. tabacum* and *N. alata*). However, their concentrations were substantially lower than those of ABA itself (10-fold on average), and no clear trends in their dynamics across pollen development were observed that could be generalized.

Jasmonates were detected in a wide range of concentrations during pollen development in all four tobacco species (Figures 5, 6 and Supplementary Tables 1–4). Jasmonic acid (JA) and its active conjugate, JA-isoleucine (JA-Ileu), represented the most conspicuous derivatives. In addition, small amounts of the JA precursor, *cis-*(C)-12-oxo-phytodienoic (*c*OPDA), were found at earlier developmental stages of *N. langsdorffii* and *N. mutabilis*. In *N. tabacum*, total jasmonate levels ranged from 705.44 (microspores; stage 1) to 41619.42 pmol/g DW (middle bicellular pollen; stage 4). The concentration of JA significantly exceeded that of JA-Ileu at the earlier developmental stages, with the maximum JA values recorded at stage 4 (25511.39 pmol/g DW). In contrast, a progressively increasing accumulation of JA-Ileu was observed until stage 6 (30795.9 pmol/g DW) (Figure 5C).

The highest amounts of jasmonates in the pollen of the other three tobacco species were detected at stage 4 (Figures 6G–I). The maxima (from 215176.3 in *N. alata* to 564432.26 pmol/g DW in *N*. *mutabilis)* were caused mainly by JA-Ileu concentrations significantly exceeding the value of JA (3- to 61-fold in *N. alata* and *N. langsdorffii*, respectively).

To sum up, the dynamics in the accumulation of stress hormones (ABA-types and jasmonates) during pollen ontogeny differed significantly between *N. tabacum* and the other three species (*N*. *alata*, *N. langsdorffii*, and *N. mutabilis*).

#### Phenolic Compounds

Three phenolic compounds, SA, BzA, and PAA, were abundant in the pollen of all four tobacco species, with extremely high levels in *N. alata* (Figures 5, 6 and Supplementary Tables 1–4). In all, BzA concentrations substantially exceeded SA and PAA throughout ontogeny.

The dynamics of each of the three phenolics differed significantly during pollen development in *N. tabacum*. While SA appeared in relatively high amounts from stage 1 to stage 5 (in the range 5796.62 and 6895.33 pmol/g DW) and then decreased at stage 6 (about 3.5-fold), the accumulation of PAA gradually increased (from 219.54 at stage 1 to 4656.99 pmol/g DW at stage 6), and BzA clearly peaked at stage 4 (312682.51 pmol/g DW) (Figures 5D–F). In contrast, the same trends in the dynamics of phenolic compounds with concentration maxima at stage 4 were observed in the pollen of the other three tobacco species (Figures 6J–R). Interestingly, PAA, which can also be categorized as a weak non-indole auxin, had similar dynamics to the indole auxins IAA and OxIAA in *N. alata*, *N. langsdorffii*, and *N. mutabilis* pollen (Figures 6A–C,M–O).

Overall, the dynamics of the analyzed phenolic compounds across pollen development, analogous to growth and stress hormones, were significantly different between *N. tabacum* and the other three tobacco species.

## Discussion

The function of phytohormones in the control of seed plant growth and development is well known (Taiz and Zeiger, 2002), but there is limited knowledge (e.g., Hirano et al., 2008; Song et al., 2013) about their involvement in pollen development. In this work, a number of phytohormone groups were detected in developing pollen grains in four *Nicotiana* species. We characterized the profiles and dynamics of specific phytohormone forms in individual pollen developmental stages to postulate how they might be involved in regulating male gametophyte development.

Following the work of Tupý et al. (1983), we recognized six developmental stages in *Nicotiana tabacum* pollen. In contrast, only five developmental stages of pollen-based morphological characteristics of flowers, organelle development and starch content were distinguished in *N. alata, N. langsdorffii*, and *N. mutabilis* (Figure 1). The differences in pollen ontogeny between *N. tabacum* and the other three tobacco species reflect a different phylogeny of the genus *Nicotiana*. This genus, comprising approximately 75 species, 40 diploids, and 35 allopolyploids, displays a range of diverse genomic changes (including gene conversion, tandem and dispersed sequence evolution, intergenomic translocations, dysploidy, polyploidy, etc., Clarkson et al., 2004). Whereas *N. tabacum* belongs to section *Nicotiana* with a basic chromosome number *n* = 24, the other species analyzed in our study belong to section *Alatae* with *n* = 10 (Clarkson et al., 2004). Similarly, there is a slight shift during pollen ontogeny in other *Nicotiana* species, such as *Nicotiana benthamiana* (*n* = 16) from section *Suaveolentes* (Steinbachová et al., in preparation).

Endogenous phytohormones and their dynamics were closely related to pollen ontogenesis in all four tobacco species. We found that CKs, auxins and ABA-type derivatives were present mainly in bioactive forms throughout pollen development (Figure 2). This confirms that pollen ontogenesis is a highly active process (Bedinger, 1992) under hormonal control.

Transcriptome analyses in pollen of *Arabidopsis* (Honys and Twell, 2004) and *N. tabacum* (Bokvaj et al., 2015) showed that all major components of the core cell cycle machinery are expressed at stage 1, the microspores, and their expression generally decreases during pollen development. However, phytohormone-related genes are expressed in a continuously increasing manner during pollen maturation (Záveská Drábková et al., in preparation), which is analogous to the concentration dynamics of numerous hormone derivatives shown here. Moreover, many transcripts have been found to persist in mature pollen (Chambers and Shuai, 2009; Grant-Downton et al., 2009). In this work, the levels of some hormones such as 2MeSZR, JA-Ileu and PAA in *N. tabacum* showed a similar trend, increasing progressively and reaching maxima at stage 6.

In general, there are two prominent maxima in the sum of phytohormones in all four *Nicotiana* species during their pollen development, a major one at stage 4 and a minor one at stage 2 (Supplementary Figure 1). These two maxima correspond well with the beginning and the end of the accumulation of starch and other material reserves. As for the differences in pollen ontogeny, the onset of starch accumulation is later in *N. tabacum* compared to *N. alata*, *N. langsdorffii*, and *N. mutabilis* (Figure 2). Moreover, the transition from polarized microspores (stage 1) after asymmetric division during pollen mitosis I (PMI) to binucleate pollen (stage 2) represents a critical moment in male gametophyte development (Hafidh et al., 2016), which is essential for determining germ cell fate (Hackenberg and Twell, 2019).

There are few and scattered reports on the role of CKs in male gametophyte development. Hirano et al. (2008) showed that synthesis of CKs occurs preferentially during the early developmental stage of rice pollen and that bioactive CKs are deactivated by cytokinin oxidase/dehydrogenase (CKX) in the later ontogenic stages. In *N. tabacum* pollen, most classical CKs were correspondingly present at stage 1, although another smaller peak was also found at stage 4 (Figure 3). In contrast, only one CK maximum was found at stage 4 in the other three species (Figure 4). However, it is not possible to compare our results on tobacco with those of Hirano et al. (2008) on rice because only microspores and late pollen were included in their study. Relatively high contents of bioactive and transport CK forms, CK phosphates and *N*-glucosides (mainly *N*7-glucosides) and, on the other hand, the absence or lack of CK *O*-glucosides represented the most typical features of developing pollen in all four tobacco species (Figure 2). Accordingly, no CK *O*-glucosides were detected in *N. tabacum* chloroplasts (Benková et al., 1999), while CK *N*-glucosides represented the predominant CK forms in *N. tabacum* shoots (Gelová et al., 2018) and leaves (Uzelac et al., 2016).

In general, *t*Z- and iP-type CKs predominated throughout the pollen development of *N. tabacum* whereas *c*Z- and iP-types prevailed in the other three tobacco species. CKs of DZ-type were completely absent in the developing pollen of *N. langsdorffii* as well as in three stages of pollen ontogeny of *N. tabacum* and two of *N. mutabilis* (Figures 3A, 4A–C and Supplementary Tables 1–4). The absence of DZ-types has also been observed in anthers of rice (Hirano et al., 2008), and the lack of these CK-types appears to be characteristic of some evolutionarily older non-vascular plants (e.g., Stirk et al., 2003; Záveská Drábková et al., 2015; Žižková et al., 2017). In *Arabidopsis* seedlings, Hošek et al. (2020) showed that DZ-type CKs are metabolically independent of other CK metabolic pathways, indicating their low abundance (e.g., Pokorná et al., 2021) compared with iP- or *t*Z-types, which might also be related to tobacco pollen.

In addition to classical CKs, hormonal analysis of tobacco pollen ontogeny revealed high levels of CK methylthioderivatives represented by 2MeSZR in all four species and 2MeSiPR in *N. langsdorffii* and *N. mutabilis* (Figures 3F, 4P–R and Supplementary Tables 1–4). To our knowledge, the methylthiolated CKs were detected here for the first time in developing pollen grains. Their accumulation differed markedly between *N. tabacum* and the other three species. While the levels of CK methylthioderivatives increased progressively during ontogeny of *N. tabacum* with a maximum at stage 6, they peaked (analogous to classical CKs) at stage 4 in *N. alata, N. langsdorffii*, and *N. mutabilis.* This suggests increasing *t*RNA turnover at stage 4 in the latter three species, as methylthiol-type CKs are known to arise exclusively *via* the *t*RNA degradation pathway (Tarkowski et al., 2010; Gibb et al., 2020). It also agrees well with the findings of Tupý (1982) that during pollen maturation, gene expression activity decreases, mRNA dissociates from ribosomes, and the uptake capacity of pollen grains further increases, indicating active utilization of metabolites released from the degenerating tapetal cytoplasm. Kambhampati et al. (2017) reported that high levels of methylthiolated *t*Z(R) could positively affect seed filling and yield formation, which was supported by the observation that methylthiol types can interact with CK receptors to trigger growth responses in plants (Daudu et al., 2017).

Auxin plays an important role in both early and late pollen development (Cecchetti et al., 2008; Chen and Zhao, 2008). The authors suggested that auxin is preferentially synthesized in anthers of *Arabidopsis* plants. Auxin appears to be involved in the coordination of pollen development and maturation by regulating cell cycle entry, controlling dehiscence and stamen filament growth (Cecchetti et al., 2008). In addition, two auxin response transcription factors ARF6 and ARF8 have been found to act redundantly in *Arabidopsis* floral organ development, in part by inducing the production of JA or reducing the conjugation or degradation of JA, which regulates anther dehiscence and flower opening (Nagpal et al., 2005).

Determination of endogenous auxin levels during *Arabidopsis* pollen development (Dupl’áková et al., 2016) revealed relatively low concentrations of auxin metabolites (no more than 30 pmol/g FW). This is in contrast to our analyzed tobacco pollen samples, where auxin concentrations were mainly in the range of hundreds to thousands of picomoles per g FW (Figures 5A, 6A–C and Supplementary Tables 1–4). In both *Arabidopsis* and tobacco pollen, IAA, IAA-Asp, and OxIAA contributed to the total auxin pool. On the other hand, some auxin metabolites, such as OxIAA-glucose ester and the auxin precursor indole-3-acetonitrile, found in *Arabidopsis* were not detected during tobacco pollen ontogeny. These results suggest that unequal levels of endogenous auxins and the presence of specific auxin derivatives may be characteristic of pollen development in different phylogenetic plant groups.

The dynamics of auxin accumulation in pollen of selected tobacco species with maxima at the earlier stage of development (stage 2) in *N. tabacum* and at the later stage (stage 4) in the other three species seems to reflect their different phylogenetic positions and the origin as well (see below). The concentration of PAA, a non-indole phenolic compound with weak auxin activity, had the same trajectory as indole auxins (peak at stage 4) in *N. alata*, *N. langsdorffii*, and *N. mutabilis* but not in *N. tabacum* pollen. Interestingly, the identical dynamics of indole auxins and PAA accumulation have also been demonstrated in other developmental processes in plants, such as somatic embryogenesis of conifers (Vondráková et al., 2018).

Pollen desiccation is an internal stress-like process in pollen formation (Reňák et al., 2014), and pollen grains are likely exposed to water stress conditions at some point during dehydration (Pacini and Dolferus, 2019). Thus, stress hormones play an essential role in pollen development. Free ABA and proline were analyzed in intact anthers and isolated pollen grains of *N. tabacum*, and variations in their patterns during pollen maturation were evaluated (Chibi et al., 1995). In contrast to our results, no conjugated forms of ABA were detected. The maximum accumulation of free ABA throughout the anthers occurred at the mid-binucleate stage when nearly 95% of ABA was located in the pollen (Chibi et al., 1995). This ABA distribution was not observed in the microspores or mature stages of the anthers, where the sporophytic tissues yielded more ABA than the pollen (Chibi et al., 1995). In our work, ABA itself represented the predominant metabolite throughout pollen ontogeny, with concentration maxima at developmental stage 5 in *N. tabacum* and at stage 2 in the other three species, followed by deactivation products (DPA, PA) and the storage form (ABA-GE), which, however, occurred at much lower concentrations (Figures 5B, 6D–F and Supplementary Tables 1–4).

Jasmonates are important regulators in plant development and response to biotic and abiotic stresses (Wasternack and Hause, 2013). An essential role of jasmonates in stamen and pollen maturation has been shown in *Arabidopsis* (Mandaokar et al., 2006), mediated by the induction of flower-specific MYB transcription factors (Yang et al., 2007). In our study, JA and its active conjugate JA-Ileu were the most prominent jasmonates detected during pollen development in all four tobacco species (Figures 5C, 6G–I and Supplementary Tables 1–4). In *N. tabacum* pollen, the highest concentration of JA was detected at developmental stage 4, corresponding with its requirement for male fertility and many aspects of flower and pollen development (Wilson and Zhang, 2009). High levels of JA localized in rice anthers, with microspores/pollen functioning cell-autonomously, were reported by Hirano et al. (2008). For JA-Ileu, our work found a progressively increasing accumulation up to stage 6 in *N. tabacum* and a peak at stage 4 in *N. alata*, *N. langsdorffii*, and *N. mutabilis*, respectively. Biologically active JA derivatives derived from isoleucine and tyramine have also been identified in the pollen of *Pinus mugo* and *Petunia hybrida* (Knöfel and Sembdner, 1995; Miersch et al., 1998). They have been shown to inhibit pollen germination and pollen tube elongation to maintain pollen dormancy (Yamane et al., 1982; Knöfel and Sembdner, 1995).

Three phenolic compounds, SA, BzA, and PAA were detected during pollen ontogeny in all four tobacco species. Among them, SA represents an important component in the regulation of plant defense, growth, and development (Yan and Dong, 2014). Although little information exists on the role of phenolics in pollen development, Rong et al. (2016) reported the regulation of pollen tip growth by SA in the *Arabidopsis* pollen tube model system. In our study, the same trends in the dynamics of SA, BzA, and PAA with concentration maxima at stage 4 were found for *N. alata*, *N. langsdorffii*, and *N. mutabilis*. In contrast, the dynamics of the three phenolics differed significantly during pollen development of *N. tabacum*.

Analogous to CKs and auxins, the concentration dynamics of stress hormones (ABA-types, jasmonates, SA) and other phenolic compounds differed significantly during pollen development between *N. tabacum* (section *Nicotiana*) and *N. alata*, *N. langsdorffii*, and *N. mutabilis* (section *Alatae*).

A general summary of changes and schematic representation of different trends in all analyzed phytohormone levels between *N. tabacum* and the other three tobacco species is shown in Figure 7. It clearly demonstrates the same tendencies in accumulation of analyzed phytohormones for all three *Alatae* representatives with the highest levels in the late bicellular pollen (stage 4). The only exception to this generalization is ABA-types reaching the maxima in early bicellular pollen (stage 2). In contrast, the same uniformity in phytohormone dynamics does not occur in *N. tabacum* representing the section *Nicotiana* (Figure 7 and Supplementary Figure 2). The mismatched dynamics in the accumulation of endogenous phytohormones, demonstrated during pollen ontogeny between *N. tabacum* and the other three species, reflect the different phylogenetic position and origin within the genus *Nicotiana* (Clarkson et al., 2004). The section *Nicotiana* is phylogenetically younger than the section *Alatae*. Moreover, *N. tabacum* is a hybrid allotetraploid between two species differing in their phylogenetic history, *Nicotiana sylvestris* (belonging to phylogenetically younger section *Sylvestres*) and *Nicotiana tomentosiformis* (phylogenetically older section *Tomentosae*; Supplementary Figure 2). This fact should be taken into consideration when assessing different hormonal trends of selected tobacco representatives during pollen development, making the story even more complex.

## Conclusion

The profiles and dynamics of phytohormones were characterized during the process of microgametogenesis in four *Nicotiana* species (*N. tabacum*, *N*. *alata*, *N. langsdorffii*, and *N. mutabilis*). Taking advantage of advanced HPLC-ESI-MS/MS, we identified twenty to thirty endogenous hormone derivatives, including CKs, auxins, ABA, and its derivatives, jasmonates and phenolic compounds, and tracked their concentration changes throughout pollen development.

Interestingly, all three species of the section *Alatae* have shown the same trend of all measured phytohormones with concentration maxima in the late bicellular pollen (stage 4), except for ABA-types peaking in early bicellular pollen (stage 2). On the contrary, we did not reveal the same uniformity in phytohormone dynamics in *N. tabacum* representing the section *Nicotiana* (Figure 7), which strongly reflected a different origin of representatives of the two *Nicotiana* sections.

To our knowledge, this work represents the most comprehensive review of plant hormones during the process of pollen development currently available, and evidence for the involvement of certain phytohormone forms, such as *c*Z- and methylthiol-type CKs, ABA derivatives, PAA and BzA, in pollen ontogeny has been provided for the first time here.

## Data Availability Statement

The original contributions presented in the study are included in the article/Supplementary Material, further inquiries can be directed to the corresponding authors.

## Author Contributions

LZD conceived and designed the experiments. LZD, EP, and PD performed the experiments. LZD and EP analyzed the data. JK, LS, and LZD performed microscopic observations and documentation. LZD, EP, VM, and DH wrote the manuscript. All authors have read the manuscript and approved it for submission.

## Conflict of Interest

The authors declare that the research was conducted in the absence of any commercial or financial relationships that could be construed as a potential conflict of interest.

## Publisher’s Note

All claims expressed in this article are solely those of the authors and do not necessarily represent those of their affiliated organizations, or those of the publisher, the editors and the reviewers. Any product that may be evaluated in this article, or claim that may be made by its manufacturer, is not guaranteed or endorsed by the publisher.

## Supplementary Material

Supplementary Figure 1 Changes in the sum of phytohormones during pollen development of *Nicotiana tabacum* **(A)**, *N. alata* **(B)**, *N. langsdorffii* **(C)**, and *N. mutabilis* **(D)**. The total pool of phytohormones includes auxins, abscisic acid (ABA) and its derivatives, cytokinins (CKs), salicylic acid (SA), and jasmonates (JA).

Supplementary Figure 2 Summary of phylogenetic relationships of the genus *Nicotiana* diploid based on plastid and nuclear markers with hybrid origin of *Nicotiana tabacum.* Modified and adapted from Clarkson et al. (2004).

Supplementary Table 1 Concentrations (pmol/g DW) of endogenous phytohormones during six developmental stages of *Nicotiana tabacum* pollen.

Supplementary Table 2 Concentrations (pmol/g DW) of endogenous phytohormones during five developmental stages of *Nicotiana alata* pollen.

Supplementary Table 3 Concentrations (pmol/g DW) of endogenous phytohormones during five developmental stages of *Nicotiana langsdorffii* pollen.

Supplementary Table 4 Concentrations (pmol/g DW) of endogenous phytohormones during five developmental stages of *Nicotiana mutabilis* pollen.
