# Supplementary figures and images for: Hormonome Dynamics During Microgametogenesis in Different Nicotiana Species
Source: Front Plant Sci. 2021 Oct 15;12:735451. doi: 10.3389/fpls.2021.735451 (PMC8553967; doi:10.3389/fpls.2021.735451)

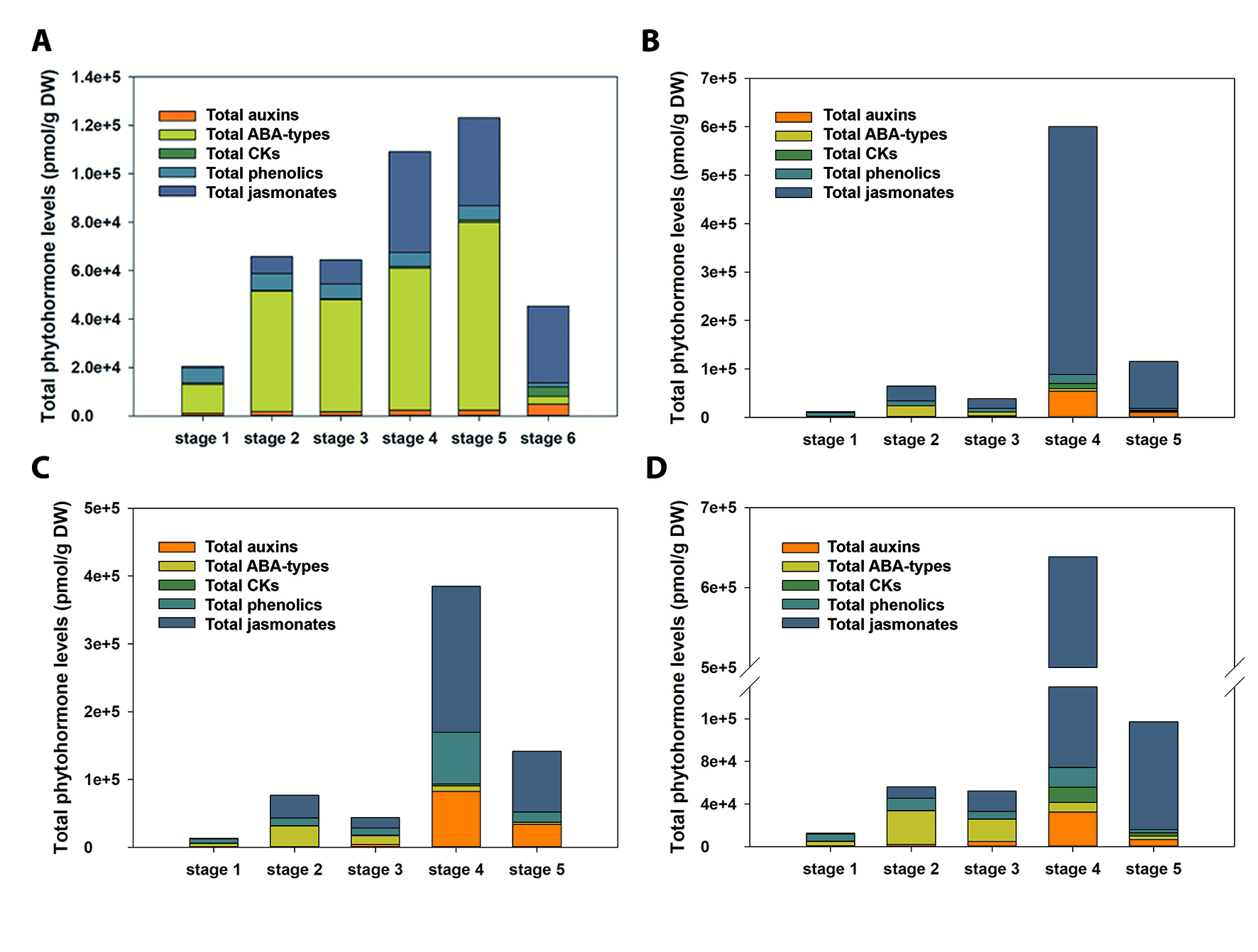

Supplement: Supplementary Figure 1 — Changes in the sum of phytohormones during pollen development of Nicotiana tabacum (A), N. alata (B), N. langsdorffii (C), and N. mutabilis (D). The total pool of phytohormones includes auxins, abscisic acid (ABA) and its derivatives, cytokinins (CKs), salicylic acid (SA), and jasmonates (JA). [file Image_1.JPEG]

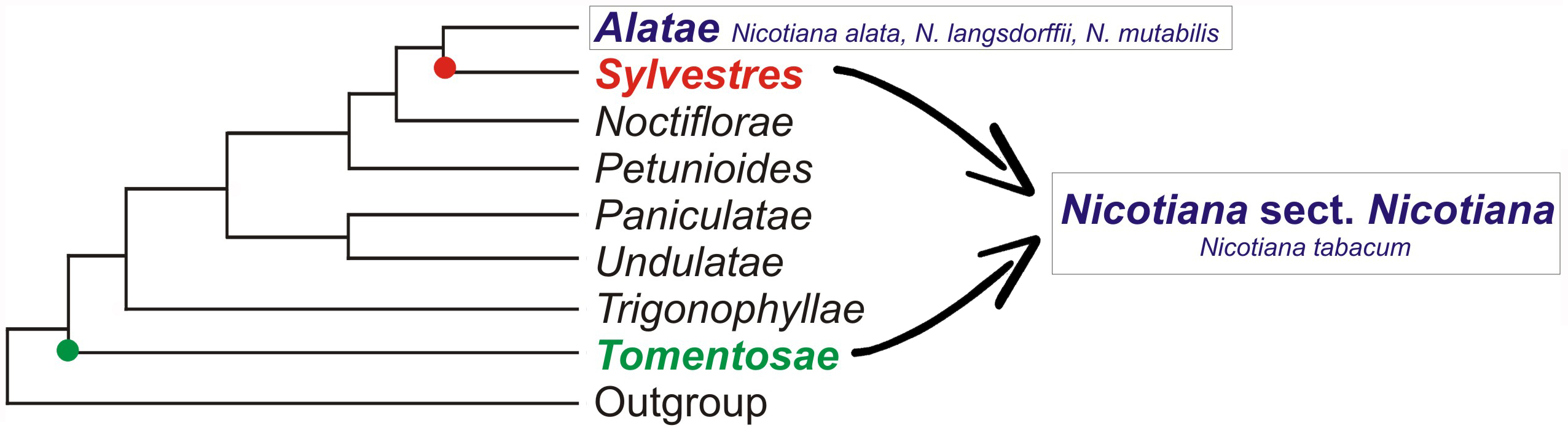

Supplement: Supplementary Figure 2 — Summary of phylogenetic relationships of the genus Nicotiana diploid based on plastid and nuclear markers with hybrid origin of Nicotiana tabacum. Modified and adapted from Clarkson et al. (2004). [file Image_2.jpg]
